# A Thermodynamic Perspective of Cancer Cells’ Volume/Area Expansion Ratio

**DOI:** 10.3390/membranes13120895

**Published:** 2023-11-30

**Authors:** Umberto Lucia, Debora Fino, Thomas S. Deisboeck, Giulia Grisolia

**Affiliations:** 1 Dipartimento Energia “Galileo Ferraris”, Politecnico di Torino, Corso Duca degli Abruzzi 24, 10129 Torino, Italy; 2 Dipartimento di Scienza Applicata e Tecnologia, Politecnico di Torino, Corso Duca degli Abruzzi 24, 10129 Torino, Italy; 3 Department of Radiology, Harvard-MIT Martinos Center for Biomedical Imaging, Massachusetts General Hospital and Harvard Medical School, 149 Thirteenth Street, Charlestown, MA 02129, USA; 4 Dipartimento di Ingegneria dell’Ambiente, del Territorio e delle Infrastrutture, Politecnico di Torino, Corso Duca degli Abruzzi 24, 10129 Torino, Italy

**Keywords:** biophysics, constructal law, irreversibility, cancer, volume/area ratio in cell systems

## Abstract

The constructal law is used to improve the analysis of the resonant heat transfer in cancer cells. The result highlights the fundamental role of the volume/area ratio and its role in cancer growth and invasion. Cancer cells seek to increase their surface area to facilitate heat dissipation; as such, the tumour expansion ratio declines as malignant cells start to migrate and the cancer expands locally and systemically. Consequently, we deduce that effective anticancer therapy should be based on the control of some ion transport phenomena in an effort to increase the volume/area ratio. This emphasises restricting the local and systemic spatial expansion of the tumour system and thus gives further credence to the superior role of novel anti-migratory and anti-invasive treatment strategies over conventional anti-proliferative options only.

## 1. Introduction

The constructal law was proposed in 1996 [1] by stating that [2] any real system’s configuration evolves by following the thermodynamic pathway that increases the access to flows responsible for the evolution itself. This statement summarises a universal law of physics [3] that expresses the evolutionary designs of flow architectures [4] both in nature and human-made systems. This law represents a unified approach to animate and inanimate systems under a thermodynamic improvement of optimisation engineering and the physical analysis of complex systems [5].

In a thermodynamic approach to biological cells, they behave as thermodynamic engines that convert the inflow heat into work [6] by using the following different cellular metabolic pathways [7]:

- The Krebs cycle, characteristic of normal cells, which is based on the oxidation of acetyl-CoA, and derived from carbohydrates, lipids, and proteins;
- The Warburg cycle, found in cancer cells, which is based on fermentation over the aerobic respiratory pathway.

Consequently, any cell dissipates heat into its environment [6,8,9]. Different metabolic processes have varying thermodynamic efficiencies [10]; as such, via the usual behaviour of open systems, the aforementioned metabolically different cycles should outflow two distinct heat fluxes through the cell membrane.

We have extended this approach to cell systems by considering the heat transfer phenomenon from a cell to its environment. Thus, fluxes are fundamental physical quantities, and the constructal law focuses on the geometrical characteristics of cancer cells. Following Zivieri et al. [11], the results of a study of the entropy variation due to heat fluxes are fundamental in the analysis of biochemical reactions in living cells [12,13].

In oncology, Larry Norton and Richard Simon introduced the fundamental Norton–Simon hypothesis, which can be expressed as follows: “Therapy results in a rate of regression in tumour volume that is proportional to the rate of growth that would be expected for an unperturbed tumour of that size” [14,15,16]. This statement suggests that tumour size has a fundamental role in cancer cells’ behaviour. Moreover, this hypothesis was recently improved using the constructal law approach, obtaining the role played by temperature, cell number density, and electrochemical affinity [17].

Still, ‘volume’ remains the interesting property of this clinical evidence.

In 1825, Benjamin Gompertz showed that cell growth (normal or cancerous) follows a characteristic curve [18]. In the early part of the Gompertz curve, cell numbers rapidly increase and mitosis seems to outweigh apoptosis, while close to the plateau phase, they are balanced. An open question remains regarding the cause of the curve’s shape behaviour and whether it is due to a reduction in mitosis or an increase in apoptosis, or other biophysical or biochemical processes that occur [19].

Norton proposed an intriguing explanation based on the geometry of a tumour [16]. In oncology, geometry is a mathematical tool to quantify the dimensions of biological systems, which are shown to be fractals [20]. This analysis is focused on the cell mass to volume ratio [16].

However, in 1931, Warburg showed that normal and cancer cells present different metabolic pathways [21,22,23,24]. Concerning the previous consideration that cells must get rid of heat, and that this heat flux is related to cell efficiency, we highlight that the fundamental geometrical characteristic of heat transfer is the area of the cell’s external surface. Thus, the constructal law can play a fundamental role in this analysis.

This paper will present an analysis of the geometrical characteristics of cancer starting from some previous results [25,26] with regards to the role of heat transfer in cancer, also considering possible anticancer therapeutic strategies.

## 2. Materials and Methods

Warburg highlighted the role of energy conversion in biosystems [21]: cells are able to convert external metabolites into living processes, while heat outflow dissipates into the cell environment [6,8]. A thermodynamic approach to cell metabolism is therefore based on the inflow of metabolites that are converted into thermodynamic useful work and wasted heat.

A thermo-kinetic lumped model has been used to model this heat transfer [25]. As heat flow is transferred by convection between the cell’s membrane and the fluids around it, then [27]:

(1) $$\dot{Q}=\rho_{cell}Vc_{cell}\frac{dT_{cell}}{dt}=\alpha A\Delta T$$

where $\dot{Q}$ represents the heat power transferred by convection, $\rho_{cell}$ is the cell mass density, *V* stands for the cell’s volume, $c_{cell}$ is its specific heat, $T_{cell}$ stands for the cell temperature, α denotes the coefficient of convection, *A* stands for the surface area of the cell, which varies during the phases of the development of the cell itself, and $\Delta T=T_{cell}-T_{env}$ depicts the temperature difference between the cell’s temperature and that of the environment. Thus, the lumped model has a characteristic time that can be defined [25] as:

(2) $$\tau =\frac{\rho \;c}{\alpha }\;\frac{V}{A}$$

where ρ stands for the density of the cell, *c* is its specific heat, α is the convective coefficient, *V* is the cell volume, and *A* denotes its surface area. Of course, a cell is not able to change ρ, *c*, or α quickly. However, a cell can relatively abruptly change the V/A ratio. The characteristic time constant τ represents a measure of the response of a thermal system to a thermal interaction; in particular, in the case of cells, it is related to the ability of a cell to export heat. Thus, if we introduce the basis of the constructal law, i.e., maximum heat flux, it follows that:

(3) $$\dot{Q}=\alpha \;A\;\Delta T=\alpha \;A\;\Delta T_{0}\;e^{-t/\tau }$$

The thermal transient process ends after around t=3τ, when the value of the final temperature difference is considered around 5% of the initial value [28]. Thus, the hypothesis is that heat is at a maximum in relation to the cell surface area *A* and the characteristic time τ at the end of the transient interval (t=3τ):

(4) $$\delta \dot{Q}\geq 0\Rightarrow e^{-t/\tau }\;dA+A\;e^{-t/\tau }\;t\;\frac{d\tau }{\tau^{2}}\geq 0$$

Considering:

(5) $$d\tau =\frac{\rho c}{\alpha }\;\left(\frac{1}{A}\;dV-V\;\frac{dA}{A^{2}}\right)$$

we can obtain:

(6) $$\frac{dA}{A}\leq \frac{3}{2}\;\frac{dV}{V}\Rightarrow \ln\left(1+\frac{\Delta A}{A}\right)\leq \ln{\left(1+\frac{\Delta V}{V}\right)}^{3/2}\Rightarrow \frac{\Delta A}{A}≲\frac{3}{2}\;\frac{\Delta V}{V}$$

where ΔA and ΔV represent the variation in the surface area and in the volume, respectively.

## 3. Results

The resulting Equation (6) can be interpreted as follows: for normal, non-cancerous cells, in support of single-cell growth, the relative variation in its surface area must be lower than 1.5 times the relative variation in its volume, otherwise growth is impeded.

This result agrees with the measurements of the surface-area-to-volume ratio determined in an analysis of the cellular viscoelasticity of red blood cells, which was experimentally determined as 1.50±0.12 [29].

Moreover, the relationship between morphology and phenotype has been recently investigated, showing that changes in cell shape precede and trigger some modifications in gene expression and enzymatic function, but also in the tumour metabolome. In particular, MCF-7 and MDA-MB-231 breast cancer cells were analysed, showing that relevant transitions of the tumour metabolome follow the reorganisation of the cell membrane architecture induced by environmental cues [30], suggesting cells undergo a phenotypic reversion [31].

An example is reported in ref. [16] of mouse breast ductal tree normal and cancer cells. Norton highlighted that normal cells present a fractal dimension of 2.5, while cancer cells have a fractal dimension of 2.1 [16]. However, when the cancer is spatially aggressive, Norton has shown that the fractal dimension in infiltrating ductal adenocarcinoma is greater than that in normal breast tissue, so an adenocarcinoma might have a high fractal dimension of say 2.98 (>1.5). This is very dense compared to the dimension of 2.25 of normal breast tissue [16]. These results also confirm the change in behaviour of tumours before they transform and become more malignant [32].

## 4. Discussion and Conclusions

The dynamic equilibrium between cell proliferation and cell death, tissue morphogenesis, and differentiation is the control process of normal cell behaviour [33]. When this balance is lost, transformation may occur and cancer can evolve—a combined effect of dysregulated morphogenesis and uncontrolled proliferation [33]. The mechanisms that generate cancerous growth may be genetic or epigenetic but may also result from changes in the cell’s environment and tissue organisation. Many processes are related to the nano-mechanical properties of the cell membrane and its structure [34].

In this context, cell surface modifications may confer therapeutic potential. New drug carriers can be designed more efficiently for drug delivery barriers at every level of drug distribution, including systemic, tissue, and cellular levels [35]. Indeed, in cells, all biological processes convert different forms of molecular energy into mechanical work, which often affects conformational changes and displacements [35].

The analysis of ion fluxes in cancer highlighted [36,37,38] the regulatory role of ion channels and transporters and its consequences on cell cycle phases of neoplastic progression, resistance to apoptosis, and tumour cell invasion [39].

Cancer cells can store less chemical energy than normal cells due to their lower efficiency to metabolise energy [10]; over the same time interval, a cancer cell needs to export more of this unused energy in the form of heat towards the cell’s environment. It follows that cancer cells need to increase their external surface to optimise the convective thermal fluxes. As a biomechanical consequence, on the single-cell level, such a surface extension is reflected in cell elongation, which in turn could trigger the onset of cell motility [32]. On a multicellular scale, such tumour invasion should damage the adjacent tissue architecture and thus reduce mechanical confinement, which, conceptually, facilitates further volumetric on-site growth and fuels a vicious cycle. We stress that related biochemical phenomena occur; indeed, Na^+^, K^+^, and Cl^−^ ion fluxes determine membrane voltage regulation, while Ca^2+^ and Mg^2+^ ion fluxes control protein folding and Zn^2+^ controls HCO^3−^ formation. Each ion induces different heat effects, but all of them generate heat loss. All these processes occur simultaneously during cancer growth and metastasis, and cancer growth is a result of cell’s energy management and of heat and mass transport through the cancer cell’s membrane. Thus, cancer cells must strive to decrease their “volume/area ratio”. This would explain the transition to the aforementioned migratory phenotype on the single-cell level as well as angiogenesis on the multicellular tumour level, where the sprouting of vessels towards the tumour expands the exchange surface as much as it further reduces confinement and provides routes for cancer cell dissemination and eventually metastasis. This can be understood as a systemic tumour area optimisation [32] constrained by the ‘host’ organ’s specific carrying capacity [32]. Intriguingly, highly metastatic kidney cancer cells have been shown to exhibit increased cytoskeletal dynamic and deformation, which are related to processes of vascular invasion [40].

Consequently, novel concepts for anticancer therapy could be based on the control of some ion transport phenomena in an effort to target the tumour system’s intrinsic ‘volume/area’ ratio decrease. Specifically, this points towards favouring anti-migratory and anti-invasive therapeutic strategies as opposed to the conventional anti-proliferative modalities, which would reduce the numerator disproportionally and thus unintentionally create (re-)growth-promoting conditions. This result is supported by studies of the thermophysical behaviour of proteins; indeed, it has been shown that the temperature relaxation of proteins can be described using a macroscopic approach, but also that the protein–water interface plays a fundamental role in the thermal relaxation of biomolecules [41], with numerical and experimental evidence that interfacial heat transfer is strictly related to the size of the system [42].

Lastly, a numerical confirmation of our conclusion can be obtained by considering that the electric membrane potential is expressed as:

(7) $$\Delta \phi =H-T_{0}\;\dot{Q}\;\tau -2.3\;\frac{RT_{0}}{F}\;\Delta (pH)$$

where ϕ denotes the cell’s electric membrane potential, $T_{0}$ is the environmental temperature of the cell, $\dot{Q}$ stands for the heat power flow, τ represents the characteristic time as in Equation (2), *R* and *F* are the universal ideal gas and the Faraday constants, respectively, and ΔpH is the variation in the pH between the two membrane surfaces. Analysing breast cancer, and comparing normal and tumoural cells, Norton [16] highlighted that the density of the tissue is preserved for longer, so that more cells per unit of volume contribute to the growth. In addition, we consider that the biological matter of cancer and normal cells is composed of the same soft matter, introducing the hypothesis that, in first approximation, we can consider the thermophysical properties of cancer and normal cells as *equal*. As such, we keep all quantities constant, except τ. It then follows that:

(8) $$\frac{\Delta \phi_{\mathrm{cancer}}-\Delta \phi_{\mathrm{normal}}}{\Delta \phi_{\mathrm{normal}}}=\frac{\tau_{\mathrm{cancer}}-\tau_{\mathrm{normal}}}{\tau_{\mathrm{normal}}}=\frac{{\left(V/A\right)}_{\mathrm{cancer}}-{\left(V/A\right)}_{\mathrm{normal}}}{{\left(V/A\right)}_{\mathrm{normal}}}$$

which, considering characteristic values [43,44,45] of Δϕ for cancer (−10 mV) and normal cells (−70 mV), respectively, yields the following result:

(9) $${\left(\frac{V}{A}\right)}_{\mathrm{cancer}}=\frac{1}{7}{\left(\frac{V}{A}\right)}_{\mathrm{normal}}$$

Equation (9) proves that an expanding cancer cell or multicellular tumour always has a lower volume/area ratio in relation to that of normal cells or tissue.

Finally, we note that our approach does not neglect other metabolic levers that tumours can utilise. For instance, Equation (3) contains another fundamental thermophysical quantity: the convection coefficient α. Tumours can increase this coefficient to further increase heat transfer and this is related to blood flow. Thus, tumour-induced angiogenesis not only increases nutrient availability and further reduces mechanical confinement, but also aids thermal outflow. In reality, these and other factors work in parallel, and our approach accounts for them conceptually.

## Data Availability

The data used have been here evaluated and the reference to literature is quoted in the References.

## References

[1] Bejan A. (1996). Street Network Theory of Organization in Nature. J. Adv. Transp..

[2] Bejan A. (1997). Constructal-Theory Network of Conducting Paths for Cooling a Heat Generating Volume. Int. J. Heat Mass Transf..

[3] Bejan A., Lorente S. (2013). Constructal law of design and evolution: Physics, biology, technology, and society. J. Appl. Phys..

[4] Bejan A. (2023). Constructal design evolution versus topology optimization. Int. Commun. Heat Mass Transf..

[5] Bejan A. (1997). Advanced Engineering Thermodynamics.

[6] Katchalsky A., Kedem O. (1962). Thermodynamics of Flow Processes in Biological Systems. Biophys. J..

[7] Voet D., Voet J.G. (2004). Biochemistry.

[8] Schrödinger E. (1944). What is Life? The Physical Aspect of the Living Cell.

[9] Callen H.B. (1960). Thermodynamics.

[10] Lucia U. (2012). Irreversibility in biophysical and biochemical engineering. Phys. A Stat. Mech. Its Appl..

[11] Zivieri A., Pacini N., Finocchio G., Carpentieri M. (2017). Rate of entropy model for irreversible processes in living systems. Sci. Rep..

[12] Zivieri A., Pacini N. (2017). Is an Entropy-Based Approach Suitable for an Understanding of the Metabolic Pathways of Fermentation and Respiration?. Entropy.

[13] Zivieri A., Pacini N. (2018). Entropy Density Acceleration and Minimum Dissipation Principle: Correlation with Heat and Matter Transfer in Glucose Catabolism. Entropy.

[14] Norton L., Simon R. (1977). Tumor size, sensitivity to therapy, and design of treatment schedules. Cancer Treat. Rep..

[15] Norton L., Simon R. (1986). The Norton-Simon hypothesis revisited. Cancer Treat. Rep..

[16] Norton L. (2005). Conceptual and Practical Implications of Breast Tissue Geometry: Toward a More Effective, Less Toxic Therapy. Oncologist.

[17] Lucia U., Ponzetto A., Deisboeck T.S. (2016). Constructal approach to cell membranes transport: Amending the ‘Norton-Simon’ hypothesis for cancer treatment. Sci. Rep..

[18] Piccart-Gebhart M.J. (2003). Mathematics and oncology: A match for life?. J. Clin. Oncol..

[19] Lucia U., Ponzetto A., Deisboeck T.S. (2015). A thermodynamic approach to the ‘mitosis/apoptosis’ ratio in cancer. Phys. A Stat. Mech. Its Appl..

[20] Cross S.S. (1997). Fractals in pathology. J. Pathol..

[21] Warburg O., Wind F., Negelein E. (1927). The metabolism of tumors in the body. J. Gen. Physiol..

[22] Weinhouse S. (1956). On respiratory impairment in cancer cells. Science.

[23] Warburg O. (1956). On the Origin of Cancer Cells. Science.

[24] Burk D., Schade A.L. (1956). On respiratory impairment in cancer cells. Science.

[25] Lucia U., Grisolia G. (2022). Thermal resonance in living cells to control their heat exchange: Possible applications in cancer treatment. Int. Commun. Heat Mass Transf..

[26] Lucia U., Grisolia G. (2020). Thermal resonance and cell behaviour. Entropy.

[27] Bejan A. (2000). Shape and Structure, from Engineering to Nature.

[28] Nag P.K. (2009). Heat and Mass Transfer.

[29] Namvar A., Blanch A.J., Dixon M.W., Carmo O.M.S., Liu B., Tiash S., Looker O., Andrew D., Chan L.J., Tham W.H. (2021). Surface area-to-volume ratio, not cellular viscoelasticity, is the major determinant of red blood cell traversal through small channels. Cell. Microbiol..

[30] Lucia U., Deisboeck T.S., Ponzetto A., Grisolia G. (2023). A Thermodynamic Approach to the Metaboloepigenetics of Cancer. Int. J. Mol. Sci..

[31] D’Anselmia F., Valerio M., Cucina A., Galli L., Proietti S., Dinicola S., Pasqualato A., Manetti C., Ricci G., Giuliani A. (2011). Metabolism and cell shape in cancer: A fractal analysis. Int. J. Biochem. Cell Biol..

[32] Deisboeck T.S., Wang Z. (2007). Cancer dissemination: A consequence of limited carrying capacity?. Med. Hypotheses.

[33] Muthuswamy S.K., Xue B. (2012). Cell Polarity As A Regulator of Cancer Cell Behavior Plasticity. Annu. Rev. Cell Dev. Biol..

[34] Pelling A.E., Sehati S., Gralla E.B., Valentine J.S., Gimzewski J.K. (2004). Local nanomechanical motion of the cell wall of *Saccharomyces cerevisiae*. Science.

[35] Bustamante C., Chemla Y.R., Forde N.R., Izhaky D. (2004). Mechanical Processes in Biochemistry. Annu. Rev. Biochem..

[36] Arcangeli A., Crociani O., Lastraioli E., Masi A., Pillozzi S., Becchetti A. (2009). Targeting ion channels in cancer: A novel frontier in antineoplastic therapy. Curr. Med. Chem..

[37] Becchetti A., Munaron L., Arcangeli A. (2013). The role of ion channels and transporters in cell proliferation and cancer. Front. Physiol..

[38] Becchetti A. (2011). Ion channels and transporters in cancer. 1. Ion channels and cell proliferation in cancer. Am. J. Physiol..

[39] Becchetti A., Arcangeli A. (2010). Integrins and ion channels in cell migration: Implications for neuronal development, wound healing and metastatic spread. Adv. Exp. Med. Biol..

[40] Coughlin M.F., Fredberg J.J. (2013). Changes in cytoskeletal dynamics and nonlinear rheology with metastatic ability in cancer cell lines. Phys. Biol..

[41] Lervik A., Bresme F., Kjelstrup S., Bedeaux D., Rubi J.M. (2010). Heat transfer in protein-water interfaces. Phys. Chem. Chem. Phys..

[42] Lervik A., Bresme F., Kjelstrup S. (2009). Heat transfer in soft nanoscale interfaces: The influence of interface curvature. Soft Matter.

[43] Cone C.D. (1969). Electroosmotic interactions accompanying mitosis initiation in sarcoma cells in vitro. Trans. N. Y. Acad. Sci..

[44] Cone C.D. (1970). Variation of the transmembrane potential level as a basic mechanism of mitosis control. Oncology.

[45] Cone C.D. (1971). Unified theory on the basic mechanism of normal mitotic control and oncogenesis. J. Theor. Biol..

